# Development and Validation of an Enzyme-Linked Immunosorbent Assay for the Detection of Binding Anti-Drug Antibodies against Interferon Beta

**DOI:** 10.3389/fneur.2017.00305

**Published:** 2017-07-06

**Authors:** Kathleen Ingenhoven, Daniel Kramer, Poul Erik Jensen, Christina Hermanrud, Malin Ryner, Florian Deisenhammer, Marc Pallardy, Til Menge, Hans-Peter Hartung, Bernd C. Kieseier, Elisa Bertotti, Paul Creeke, Anna Fogdell-Hahn, Clemens Warnke

**Affiliations:** ^1^Medical Faculty, Department of Neurology, Heinrich-Heine-University, Duesseldorf, Germany; ^2^Sanofi-Aventis, Deutschland GmbH, Frankfurt am Main, Germany; ^3^Neuroimmunology Laboratory, DMSC, Department of Neurology, Rigshospitalet, Copenhagen, Denmark; ^4^Department of Clinical Neuroscience, Karolinska Institutet, Stockholm, Sweden; ^5^Department of Neurology, Innsbruck Medical University, Innsbruck, Austria; ^6^INSERM 996, University Paris-Sud, Paris, France; ^7^Merck NBE Bioanalytics, Torino, Italy; ^8^Centre for Neuroscience and Trauma, Blizard Institute, Queen Mary, University of London, London, United Kingdom; ^9^Department of Neurology, University Hospital of Cologne, Cologne, Germany

**Keywords:** multiple sclerosis, anti-drug antibodies, interferon beta, enzyme-linked immunosorbent assay, biotherapy

## Abstract

**Objective:**

To develop and validate a method for the detection of binding anti-drug antibodies (ADAs) against interferon beta (IFN-β) in human serum as part of a European initiative (ABIRISK) aimed at the prediction and analysis of clinical relevance of anti-biopharmaceutical immunization to minimize the risk.

**Method:**

A two-tiered bridging enzyme-linked immunosorbent assay (ELISA) format was selected and validated according to current recommendations. *Screening assay*: ADA in serum samples form complexes with immobilized IFN-β and biotinylated IFN-β, which are then detected using HRP labeled Streptavidin and TMB substrate. *Confirmation assay*: Screen “putative positive” samples are tested in the presence of excess drug (preincubation of sera with 0.3 µg/mL of soluble IFN-β) and percentage of inhibition is calculated.

**Results:**

The assay is precise, and the sensitivity of the assay was confirmed to be 26 ng/mL using commercially available polyclonal rabbit antihuman IFN-β in human sera as the positive control.

**Conclusion:**

An ultrasensitive ELISA for IFN-β-binding ADA testing has been validated. This will form the basis to assess anti-biopharmaceutical immunization toward IFN-β with regards to its clinical relevance and may allow for the development of predictive tools, key aims within the ABIRISK consortium.

## Introduction

Biopharmaceuticals (BPs) are increasingly used for therapy of multiple diseases, including inflammatory and autoimmune disorders. Unwanted immunogenic responses to BPs, including anti-drug antibodies (ADAs), can potentially affect clinical safety and efficacy. In patients with multiple sclerosis (MS), different interferon beta (IFN-β) formulations have been approved as disease modifying therapeutics for more than two decades and experience from historical clinical ADA testing is available (1–3). Despite the increasing number of drugs that recently became available for therapy of MS, owing the well-established long-term safety profile and some concerns with alternative treatments such as the occurrence of progressive multifocal leukoencephalopathy (4), IFN-β treatment still remains one of the first-choice options for mild-to-moderate forms of relapsing MS (5). Accurate assays for screening for ADA formation in patients treated with IFN-β thus may be important and clinically relevant tools in the evolving field of precision medicine. Consensus has been reached that patients with persistent detection of ADA with high neutralizing capacity should switch therapy to a non-IFN-β alternative (6). However, frequency of ADA positivity with neutralizing capacity can vary depending on the IFN-β formulation used and the test applied, as illustrated by a wide range of positivity spanning from 2.8 to 13.8% for IFN-β 1a i.m., and 13.3 to 68.3% for IFN-β 1b s.c. depending on the testing site in Europe (3). The varying data on positivity across countries are in part explained by a limited level of harmonization of the terms and definitions of immunogenicity at the time when the data were obtained, and by a limited standardization of the assays used for ADA testing. This also refers to the data available from pivotal clinical studies relevant for drug registration (6). Within the “Innovative Medicines Initiative” (IMI^1^) funded ABIRISK consortium [Anti-Biopharmaceutical (BP) Immunization Prediction and Clinical Relevance to Reduce the Risk^2^], formed by clinicians, academic scientists, and European Federation of Pharmaceutical Industries and Associations (EFPIA) members, standardized terms and definitions have recently been published (7). The aim of this study was to develop and validate a method for the detection of ADA toward IFN-β in human serum, following the published ABIRISK recommendations. These recommendations include a typical three-tiered approach, in which the samples are first screened and confirmed for binding ADA in a two-step analysis as described in this manuscript, and then further analyzed for neutralizing capacity and titers in a bio-assay recently published (8). The assay validation set forth in this article followed established industry and regulatory guidelines (9–13).

## Materials and Methods

### Origin of Human Serum

A human serum mixed gender pool from 50 healthy individuals (Sera Laboratories International Ltd., UK) was used (neat sera). Individual human serum samples for cut-point determination were obtained from 10 healthy donors and 40 IFN-β treatment naïve MS patients. All sera were stored at −20°C until use.

### Statistical Analysis

Assay data reflecting variability are expressed in mean, sample variances or SD, and coefficient of variation (CV). A CV of ≤30% was selected as the maximum acceptable intra-plate variation between duplicates. Curve fitting and statistical analyses were performed using Excel software (Microsoft^®^), GraphPad Prism version 6 (GraphPad Software Inc., USA), and JMP (SAS, USA).

### Assay Principle

**Figure 1:**
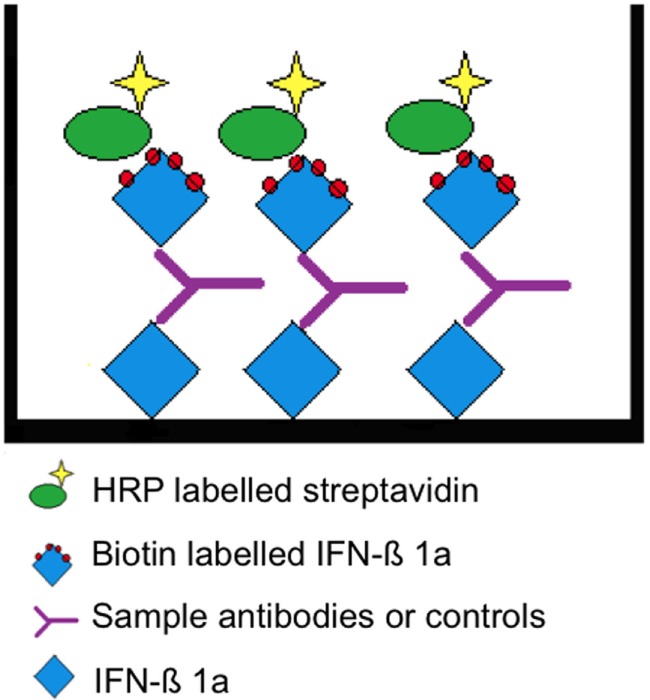
Assay principle. Interferon beta (IFN-β) 1a is non-specifically bound to the surface allowing the presentation of multiple epitopes. A “bridge” is formed by the subsequent addition of a positive serum sample or rabbit antihuman IFN-β and biotin-labeled IFN-β 1a. The latter is detected by HRP-conjugated streptavidin.

A two-tiered bridging enzyme-linked immunosorbent assay (ELISA) format is selected. All “putative positive” samples from the screening assay (tier 1) are tested in the presence of excess of drug in the confirmatory assay (tier 2). The standard assay procedure is illustrated in Table 1 and the assay principle in Figure 1.

**Table 1:** Standard assay procedure.

Microtiterplates (MTPs) are coated with 1 µg/mL soluble interferon beta (IFN-β) 1a (100 μL/well, 4°C overnight)
↓
Washing step (5 times a 300 μL/well)
↓
MTPs are blocked with blocking buffer (300 μL/well at 37°C for ≥1 h). Simultaneously, samples are diluted 1/50 in dilution buffer. Controls are prepared in matrix buffer. For the confirmatory assay, samples are spiked with soluble IFN-β 1a (1/100) and incubated at 37°C for 1 h
↓
Washing step (5 times a 300 μL/well)
↓
Samples and controls are added to MTP (100 μL/well, 37°C 1 h)
↓
Washing step (5 times a 300 µL/well)
↓
Biotinylated IFN-β 1a in dilution buffer are added (100 µL/well, 37°C 1 h)
↓
Washing step (5 times a 300 μL/well)
↓
HRP-labeled streptavidin in dilution buffer (1:10,000) is added (100 μL/well, 37°C 1 h)
↓
Washing step (5 times a 300 μL/well)
↓
TMB is mixed 1:1 (100 μL/well, room temperature 20 min, in the dark)
↓
Sulfuric acid is added (100 µL/well)
↓
Optical densities of MTPs are measured at 450 nm

The assay is performed in 96-well microtiterplates (MTPs, costar EIA/RIA Plates, Thermo Scientific Inc., USA). These plates are coated with 1 µg/mL IFN-β 1a (Avonex, Biogen, USA) in blocking buffer (0.05 M bicarbonate buffer, Pierce Perbio, USA) and incubated overnight at 4°C. MTPs are then washed five times with 300 µL PBS-tween 20 (PBS-T, Calbiochem by Merck KGaA, Germany). For all washing steps, a plate washer (Columbus plus, Tecan Group LTC, Switzerland) is used. Non-specific binding is prevented by blocking buffer (PBS-T plus 3% Albumin fraction V, Carl Roth GmbH, Germany) applying 300 µL/well for 1 h at 37°C. Samples, or positive control (PC, rabbit antihuman IFN-β, PeproTech Inc., USA) are prepared by diluting them to minimal required dilution (MRD, 1 in 50) in dilution buffer (1% BSA in PBS-Tween) or matrix buffer (1% BSA in PBS-T + neat sera 1 in 50). Non-specific binding and false positive samples are eliminated in the confirmatory assay (tier 2): samples are spiked with excess drug (IFN-β 1a 0.3 mg/mL, freeze-dried Avonex, Biogen, USA Idec Ltd., UK) and incubated for 1 h at 37°C. After washing off the blocking solution, native samples at MRD and spiked samples are added to the plate (100 μL/well) and incubated for 1 h at 37°C. After another washing step, biotinylated IFN-β 1a (Avonex, Biogen, USA; degree of labeling 8 molecules of biotin to 1 molecule IFN-β at Microcoat Biotechnologie GmbH, Germany) is added in a dilution of 1 in 1,000 at 100 µL/well and incubated for 1 h at 37°C. After washing, 100 µL/well of horseradish peroxidase-conjugated streptavidin (Strep-HRP, 1 in 10,000, Thermo Fisher Scientific Pierce Technology, USA) is added. The reagent is incubated for 1 h at 37°C. In a final washing step, unbound reagent is washed off and the substrate tetramethylbenzidine (TMB, KPL, Kirkegaard & Perry Laboratories Inc., USA) 100 µL/well is added. The TMB is incubated for 20 min at room temperature (RT); the reaction is stopped by addition of sulfuric acid 0.5 mol/L (Carl Roth GmbH, Germany). Optical densities of formed complexes are measured at 450 nm.

### Assay Development and Optimization

The optimal assay conditions and key assay parameters were established prior to starting the validation. Key reagents such as IFN-β coating concentration, biotinylated IFN-β concentrations, and streptavidin-labeled HRP dilutions were tested. The rabbit antihuman IFN-β used as PC was assessed in dose–response curves. In addition, the MRD was set up. The MRD is the minimum dilution necessary for the detection of ADA in biological matrix with least interference. To determine the MRD, five serum samples from healthy individuals were serially diluted to achieve different serum backgrounds and spiked with high PC (HPC, rabbit antihuman IFN-β at 1 µg/mL) and low PC (LPC, rabbit antihuman IFN-β at 26 ng/mL). Subsequently, the mean signal and SD for each serum dilution (*S*) and assay blank (*B*) were determined, and the *Z* factor for the HPC and LPC in each serum dilution was calculated according to the following equation (14):

$$Z=\frac{\text{[mean(}S\text{)}-\text{3SD(}S\text{)]}-\text{[mean(}B\text{)}+\text{3SD(}B\text{)]}}{\text{mean(}S\text{)}-\text{mean(}B\text{)}}$$

The *Z* factor is an estimate of the signal to noise ratio with an accepted range from 0.5 to 1.0 (14), where higher values are preferred. Based on optimal *Z* factor for the HPC and LPC the MRD was selected.

### Assay Validation

#### Screening Cut-Point

The screening cut-point is defined as the level of response at or above which an unknown sample is defined as “putative positive” for the presence of anti-IFN-β antibodies and below which it is defined as negative. The screening cut-point was established by analyzing 50 individual human sera samples at the defined MRD, studied by two operators in duplicates on at least three different days. OD values of duplicates with a CV above 30% were eliminated. Biological outliers were identified using the “box plot” method: all samples above the upper bound [75th percentile + 1.5 × (75th–25th percentile)] or below the lower bound [25th percentile−1.5 × (75th–25th percentile)] were removed. Additionally, analytical outliers, evaluated for each single assay run as described above, were removed from the individual run, and not considered for the cut-point calculation. Linear data and log-transformed data were analyzed for distribution and skewness using the Shapiro–Wilk normality and skewness test.

During this specific validation, a total of nine runs (operator 1 five runs, operator 2 four runs) were analyzed. Out of nine runs six passed the normality test and eight showed a skewness below 1.0. Therefore, a parametric approach (one sided, 95% confidence level) was selected to calculate the cut-point, using the following equation (11):

$$\text{screening cut-point}={\text{10}}^{\left(\text{mean}+\text{1.645}*\text{SD}\right)}$$

As outliers were removed and different sample numbers applied for each run, the SD was calculated from the weighted variance. To decide if a fixed or a floating cut-point could be used, two statistical tests were performed using the nine runs of the cut-point determination (after outlier removal): single factor ANOVA (analysis of variation) to evaluate if the means of the cut-point runs were significantly different or not; precision (CV%) of each run to assess if the CV% was equal to or below 15% (in this case, the variances were considered not to be significantly different). The ANOVA analysis illustrated that the means were significantly different when comparing all cut-point runs (*p* < 0.05). According to the precision calculations, the variances of the nine runs were not significantly different. These results indicated that a floating cut-point could be used. The negative control (NC, matrix at the MRD) was used to normalize the cut-point. Suitability of the NC was assessed by plotting the mean response of the NCs of each run versus the mean of the 50 corresponding individual sera tested in each cut-point run. The normalization factor (NF) was then calculated by applying the following equation applied to the log-transformed data:

NF=response at assay cut-point/mean response at NC

#### Specificity Cut-Point

Due to the 5% false-positive rate built into the screening cut-point, the confirmation of “true positives” among the “putative positive” samples requires the demonstration of specific binding to the drug. A putative positive sample is re-tested in the presence and absence of an excess of drug in solution. The specificity cut-point is defined as the “percent (%) inhibition” at or above which a sample is considered as “confirmed positive.” In order to establish the specificity cut-point, the 50 drug naïve serum samples used for the determination of the screening cut-point were spiked with an excess of IFN-β (0.3 µg/mL) and tested along with the corresponding non-spiked samples on at least three different days by two operators. The % inhibition for each sample relative to its non-spiked counterpart was calculated according to the following formula (11):

$$\mathrm{\%inhibition}=100\times \left[1-\left(\begin{array}{l} {\mathrm{OD}}_{450\mathrm{nm}}\mathrm{spiked sample}/\\ {\mathrm{OD}}_{450\mathrm{nm}}\mathrm{unspiked sample} \end{array}\right)\right]$$

% inhibition data were assessed for biological and analytical outliers as described above. Shapiro–Wilk test analysis showed that the linear data were closer to a Gaussian distribution and was more symmetrically distributed in comparison with the log-transformed data. Therefore, all calculations were performed with linear data. Seven out of nine runs passed the normality test and eight out of nine runs showed a skewness below 1. Therefore, a parametric approach was used to calculate the cut-point. The following equation was applied, accepting 1% false positivity (11):

specificity cut-point=mean %inhibition+2.33*SD

#### Specificity

To assess the specificity of the assay, varying IFN-β concentrations (ranging from 100 to 60,000 IU) were added to the HPC and tested until a signal at assay background was reached. Specificity was also shown by testing the inhibitory potentials of similar molecules such as IFN-alpha, IFN-gamma, and IL-2.

#### Sensitivity

The limit of detection (LOD) of the assay is defined by the lowest concentration of the PC yielding consistently a positive assay response. It was determined by testing a calibration curve (serial dilutions of the PC in pooled human serum ranging from 1,000 to 0.005 ng/mL) spanning the screening cut-point in six assay runs by two operators. The concentration of the PC yielding an assay response at the cut-point was interpolated using a 4PL-fitting model. The LOD was calculated with the following equation (aiming for a 1% failure rate):

LOD=mean concentration at cut-point+2.718*SD

#### Sensitivity Confirmation and Recovery

In order to confirm sensitivity, the concentration of the LPC (slightly below the calculated LOD) and higher concentrations of the PC were spiked into ten individual serum samples. In addition, all samples were tested in the confirmatory assay. The lowest concentration that was tested positive and showed a reduction above or at the specificity cut-point was defined as the sensitivity of the assay. In addition, recovery was evaluated by spiking the LPC and the HPC in ten different individual serum samples, assay buffer, and pool serum. In sample matrix, a recovery (relative to assay buffer) between 70 and 130% was accepted.

#### Assay Precision and Acceptance Criteria

The NC, the HPC, and the LPC were tested on three different days with three plates per day by two operators (in summary, 18 plates). Each plate included three independent batches of the controls. Mean response, SD, intra-, and inter-batch precision were calculated for each control. Based on the results of the intra- and inter-batch, precision acceptance criteria (aiming at a 1% failure rate) for the controls were determined. An upper acceptance limit for the NC was calculated to avoid that an unusual high response impairs the assay sensitivity (as the NC is used to normalize the screening cut-point) according to the following formula:

$$\mathrm{Upper limit of NC}=\mathrm{mean}+t_{0.01.\mathrm{df}}\times \mathrm{SD}\left(\mathrm{of overall statistics}\right)$$

For the HPC and the LPC, an upper and a lower acceptance limit were calculated according to the following formula:

Upper/lower limits of PCs=mean±t0.005.df×SD(of overall statistics)

#### Drug Interference

Drug interference was tested by spiking both the HPC and the LPC with increasing amounts of IFN-β until a signal below the screening cut-point was achieved. The highest amount of IFN-β that still led to a positive response of the LPC and the HPC was defined as drug tolerance level (one for the LPC and the HPC, respectively).

#### Robustness

Robustness is an indication of the reliability of an assay, assessed by the capacity of the assay to remain unaffected by small but deliberate variations in method parameters. The response of the NC, the LPC, and the HPC were assessed, and relevant assay steps (coating, blocking, sera spiking with soluble IFN-β, sample incubation, incubation with biotinylated IFN-β, incubation with Streptavidin-HRP) of the presented ELISA were tested by varying incubation times and measuring their influence. Optical densities were compared and analyzed (expressed in %) with reference to the results obtained under the standard conditions given in Table 1. Variations below 30% were accepted.

#### Stability

Short-term stability (4 and 24 h at RT), freeze–thaw stability (up to five freeze thaw cycles), and long-term stability (up to 6 months) were tested using the LPC and the HPC. For all the conditions tested and for each control, three individual batches were prepared. All data were measured in triplicates.

## Results

### Assay Development and Optimization

Optimization experiments defined optimal coating condition, reagent concentrations, and the MRD. The results are listed in Table 2.

**Table 2:** Overview of key assay parameters.

Optimization Parameter	Results
Coating concentration	1 µg/mL
Biotinylated interferon beta concentration	1 in 1,000 dilution
Streptavidin-labeled HRP	1 in 10,000 dilution
*Z* factor high-positive control (HPC)	0.90
*Z* factor low-positive control (LPC)	0.86
Minimal required dilution	1:50

**Validation parameter**	**Results**

Screening cut-point	Floating
Normalization factor for the screening cut-point	1.178
Specificity cut-point (confirmatory assay)	27.8%
Sensitivity (limit of detection)	26 ng/mL
Concentration HPC	1 µg/mL
Concentration LPC	26 ng/mL
Screening assay inter-batch precision negative control (NC)	14.3%
Screening assay inter-batch precision HPC	6.92%
Screening assay inter-batch precision LPC	14.90%
Acceptance criterion for the NC (screening assay)	Upper limit 0.111 OD
Acceptance criteria for the HPC (screening assay)	Upper limit: 3.658 OD
	Lower limit: 2.436 OD
Acceptance criteria for the LPC (screening assay)	Upper limit: 0.325 OD
	Lower limit: 0.129 OD
Recovery HPC (mean, range)	98%, 89–110%
Recovery LPC (mean, range)	96%, 86–109%
Drug tolerance HPC	150 ng/mL
Drug tolerance LPC	15 ng/mL

### Validation Results

#### Screening Cut-Point

**Figure 2:**
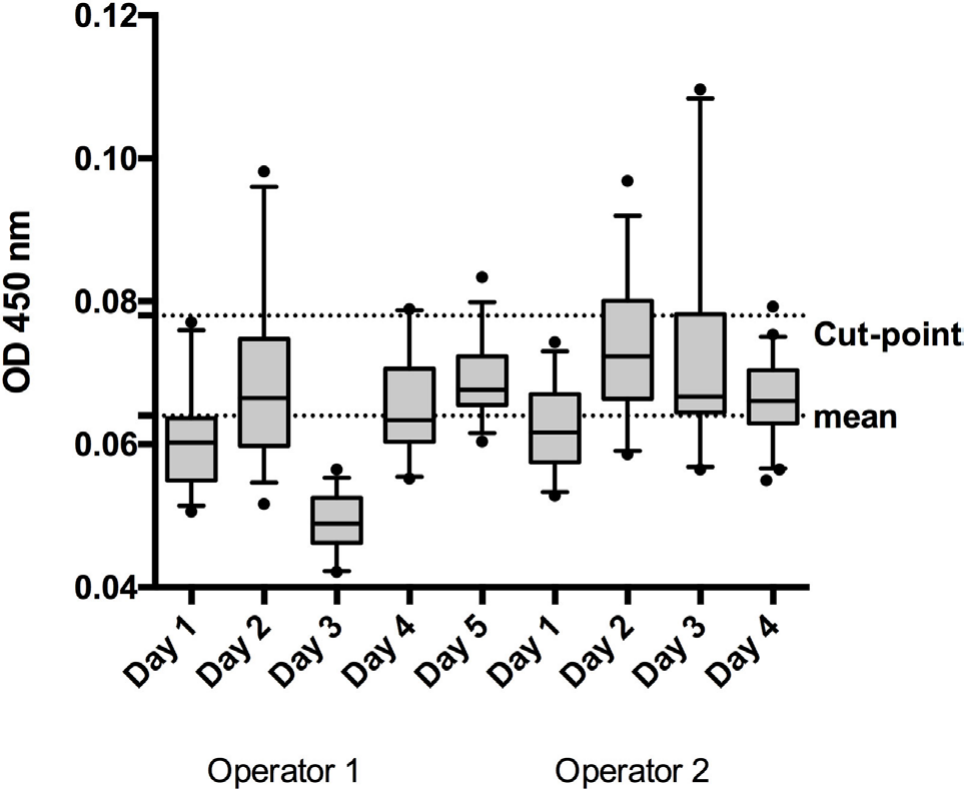
To establish the screening cut-point, 50 individual normal human sera (individual sera) were analyzed by 2 operators on 5 or 4 days, depending on the operator. The box and whisker represent the mean and 95% CI for each run.

The log-transformed values of the nine cut-point runs were more symmetric when compared with the linear data (linear: mean w value: 0.93, mean skewness absolute: 0.65; log-tranformed: mean w value: 0.95, mean skewness absolute: 0.50), thus calculations were performed on the log-scale. The mean OD in these runs was 0.064 (−1.191 for log-transformed data) (Figure 2). The pooled weighted variance was calculated to be 0.0025, resulting in a SD for cut-point calculation of 0.0503. Consequently, the cut-point was calculated as follows:

$$\text{cut-point}=\text{1}0^{\left(-1.191+1.645\text{*}0.0503\right)}=0.078\left(\text{OD}\right)$$

**Figure 3:**
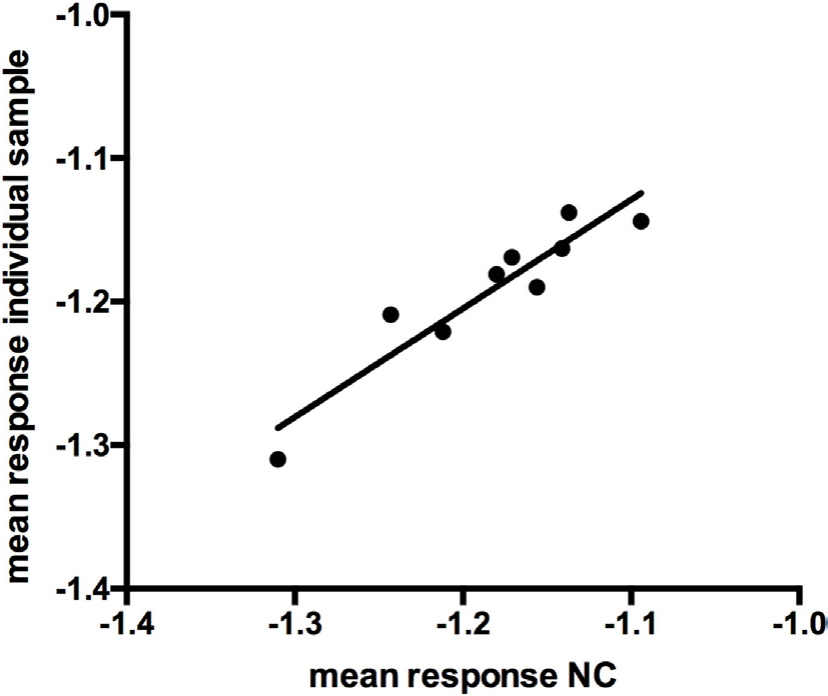
Linear fit of log-transformed OD values at negative control (NC) and individual sample response (*r* = 0.876).

The mean response for the NC (2 operators, 5 or 4 runs depending on the operator, 3 plates per run) was determined to be an OD of 0.0662. Linear fit of the mean response of the independent NCs of each run versus the mean of the individual sample response showed the suitability of the NC for use as a NF (Figure 3, *R*^2^ = 0.88). The NF was calculated to be 1.178 (0.078/0.0662). The NF multiplied by the response for the NC is used to set the plate specific, screening cut-point.

#### Specificity Cut-Point

**Figure 4:**
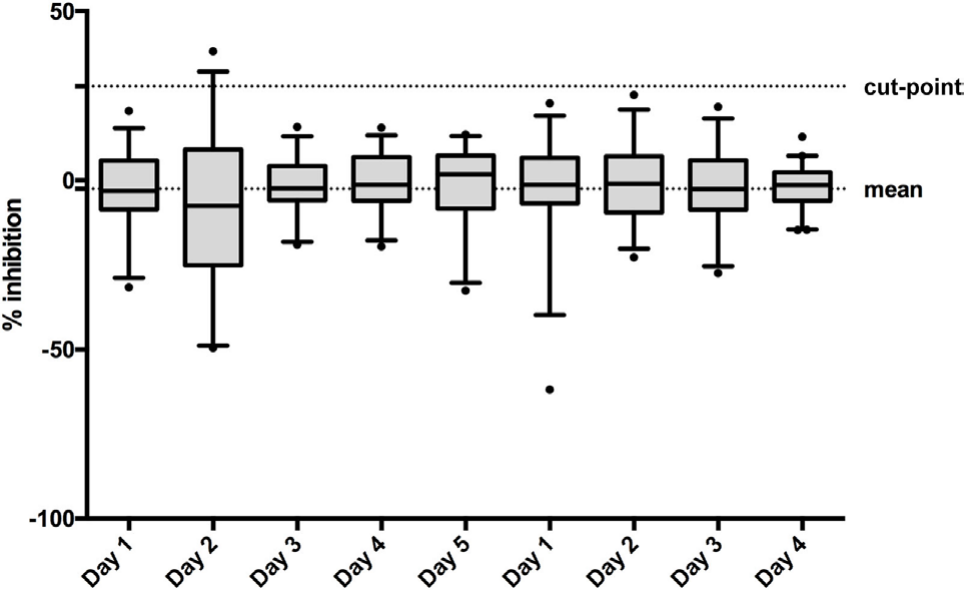
To establish the confirmatory assay cut-point, 50 individual normal human sera (individual sera) were analyzed on 5 or 4 days by two operators. The box and whisker represents the mean and 95% CI for each run.

When assessing the total mean of the percentage of inhibition in the 50 individual sera on 4/5 different days (depending the operator), linear data were more symmetric [linear: mean w value: 0.95, mean skewness (absolute): 0.45; log-transformed: mean w value: 0.87, mean skewness (absolute): 1.19], thus, all calculations were performed on the linear scale. The total mean of the percentage of inhibition was determined to be −2.52% (Figure 4). The pooled weighted variance was calculated to be 169.97%, resulting in a SD for cut-point calculation of 13.04%. Consequently, the specificity cut-point was calculated as follows:

specificity cut-point=−2.52%+2.33*13.04%=27.86%

#### Specificity

The specificity of the assay was confirmed since the soluble unlabeled concentrations of IFN-β were able to significantly inhibit the response of the HPC, while related molecules such as IFN-alpha, IFN-gamma, and IL-2 did not show a relevant inhibitory effect (Table 3).

**Table 3:** Specificity demonstrated by inhibition potential of interferon beta (IFN-β) and similar molecules.

		Inhibition (%)		
	Amount	60,000 IU	24,000 IU	100 IU
Molecule	Soluble IFN-β	94.13	93.21	18.6
	IFN-alpha	0.00	0.36	0.00
	IFN-gamma	n/a	n/a	0.81
	IL-2	n/a	4.60	0.00

#### Sensitivity

**Figure 5:**
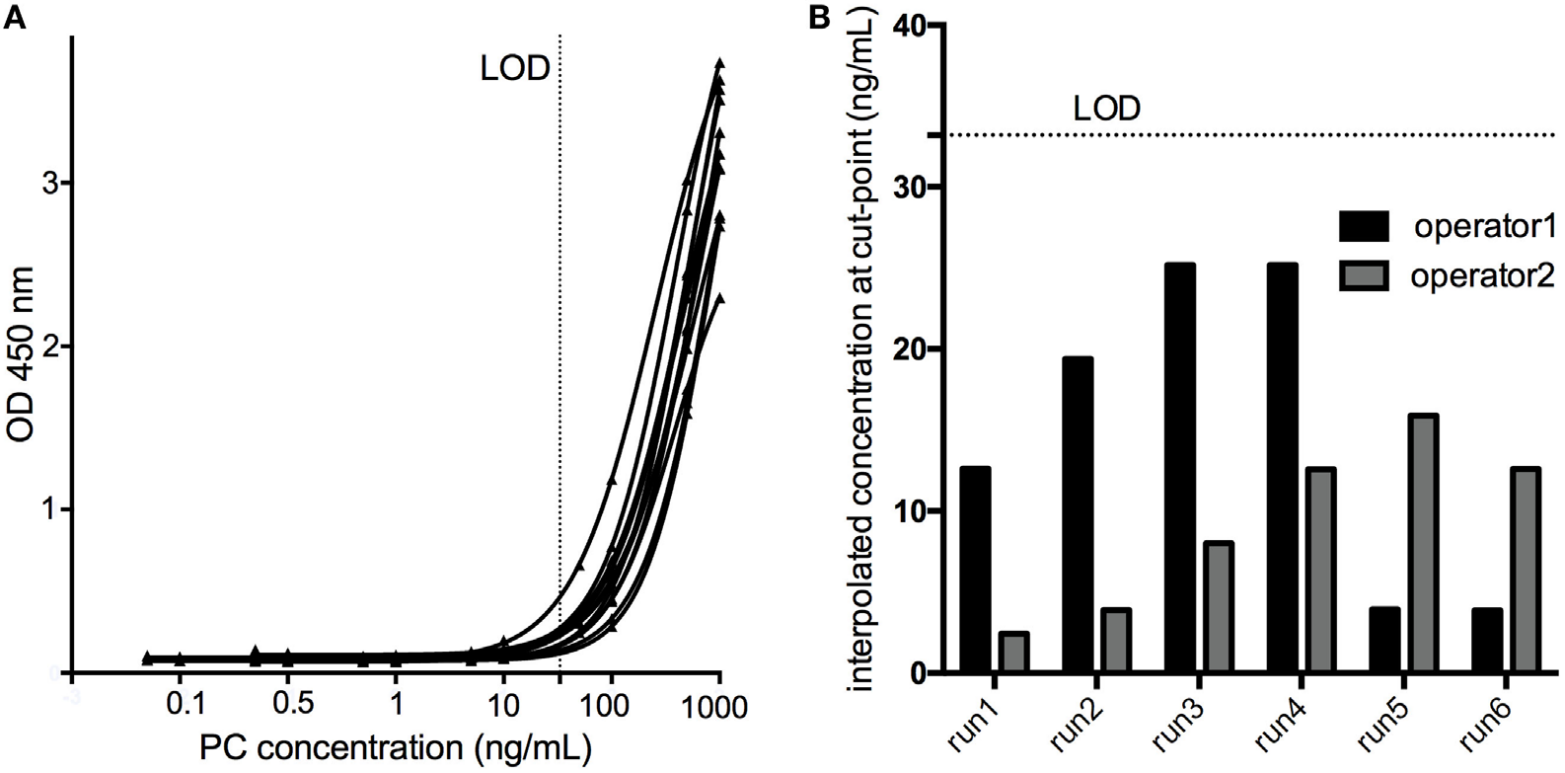
The limit of detection (LOD) of the assay was determined by testing a calibration curve [serial dilutions of the positive control (PC) in pooled human serum ranging from 1000 to 0.005 ng/mL] spanning the cut-point in six assay runs by two operators **(A)**. The concentration of the PC yielding an assay response at the cut-point was interpolated using a 4PL fitting model **(B)**, and the LOD calculated to be 33.20 ng/mL.

Six serial dilutions of the PC ranging from 1,000 to 0.005 ng/mL spanning the assay cut-point in six assay runs by two operators revealed a mean concentration at assay cut-point of 12.15 ng/mL, with a SD of 7.76 ng/mL. Consequently, the LOD (aiming at a 1% failure rate) was calculated to be 12.12 ng/mL + (2.718 × 7.76 ng/mL) = 33.20 ng/mL (Figure 5).

#### Sensitivity Confirmation and Recovery

**Figure 6:**
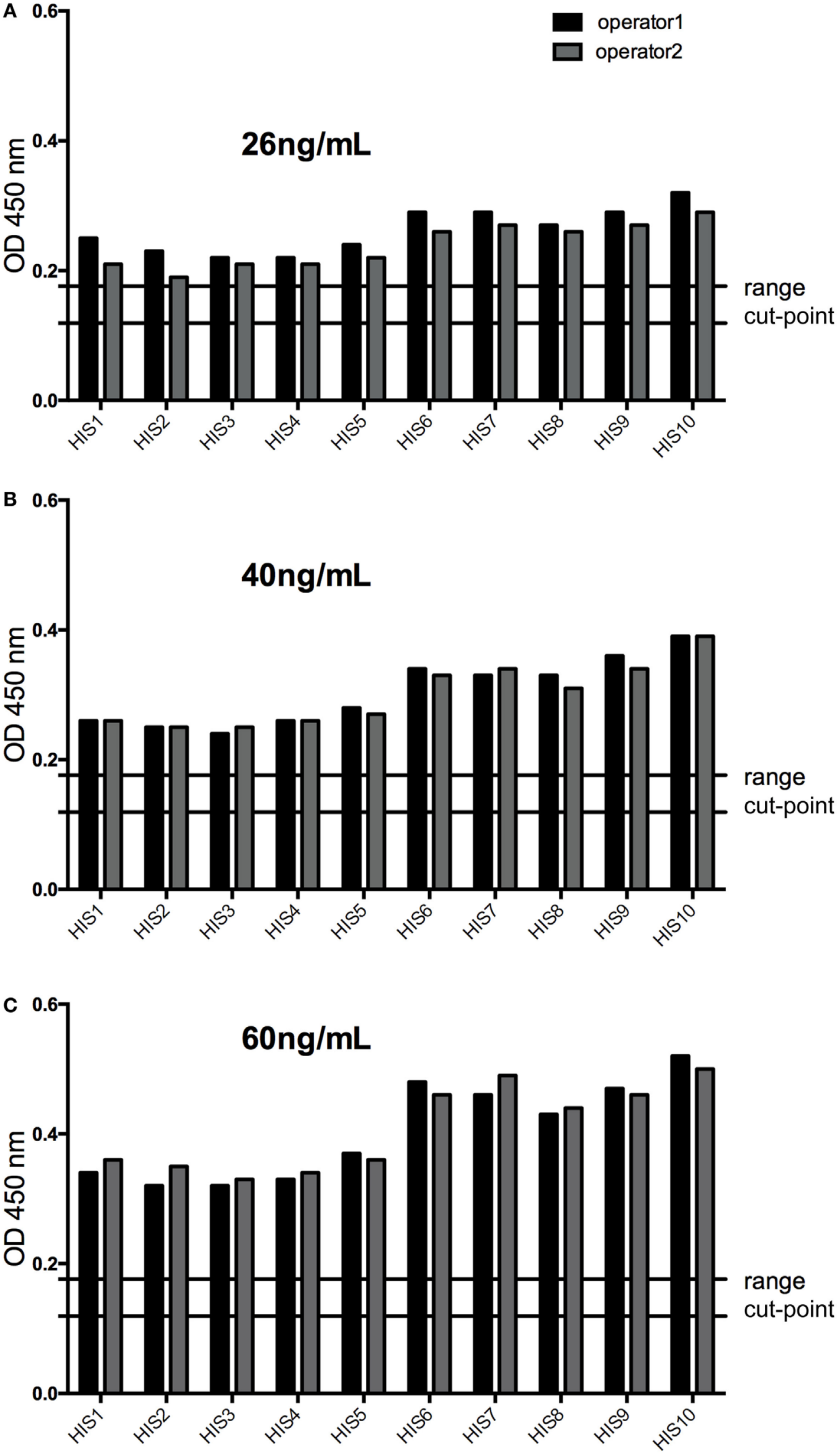
The response of low-positive control (LPC) at 26 ng/mL **(A)** was above the assay cut-point (screening and confirmation) in all individual sera tested confirming an assay sensitivity at the level of the LPC (slightly below the calculated limit of detection), as well as at 40 ng/mL, **(B)** and at 60 ng/mL **(C)** of the positive control antibody. HIS, human individual serum; cut-point range: maximal and minimal floating cut-point of the runs of this experiment.

The response of the LPC with a PC concentration slightly below the calculated LOD (selected at 26 ng/mL during assay optimization) was above the assay cut-point (screening and confirmation) in all individual sera tested, confirming an assay sensitivity at the level of the LPC. This was also shown for concentrations of the PC of 40 and 60 ng/mL (Figure 6). Furthermore, the LPC was detected positive during all the precision runs (54 values). In addition, the mean recovery tested for the HPC and the LPC did fulfill the anticipated range of 70–130% (Table 2), again supporting the determined assay sensitivity. The results also confirmed the selected MRD at 1 in 50 of the samples to be appropriate to minimize matrix effects.

#### Assay Precision and Acceptance Criteria

Precision and acceptance criteria for the controls used were assessed. For the NC, the intra-batch precision of the screening assay ranged from 2.8 to 32.0%. The inter-batch precision and the upper limit for the NC were calculated as listed in Table 2. The intra-batch precision of the screening assay for the HPC ranged from 2.1 to 17.6%, and for the LPC, from 9.8 to 31.3%. The inter-batch and the upper and lower limits of the PCs are found in Table 2. The precision of the confirmatory assay for the HPC ranged from 0.14 to 3.19%, and the acceptance criteria were calculated at 94.54–98.62% inhibition for the HPC. The precision of the confirmatory assay for the LPC ranged from 6.38 to 18.23%, and the acceptance criteria were calculated at 53.65–76.58% inhibition for the LPC.

#### Drug Interference

**Figure 7:**
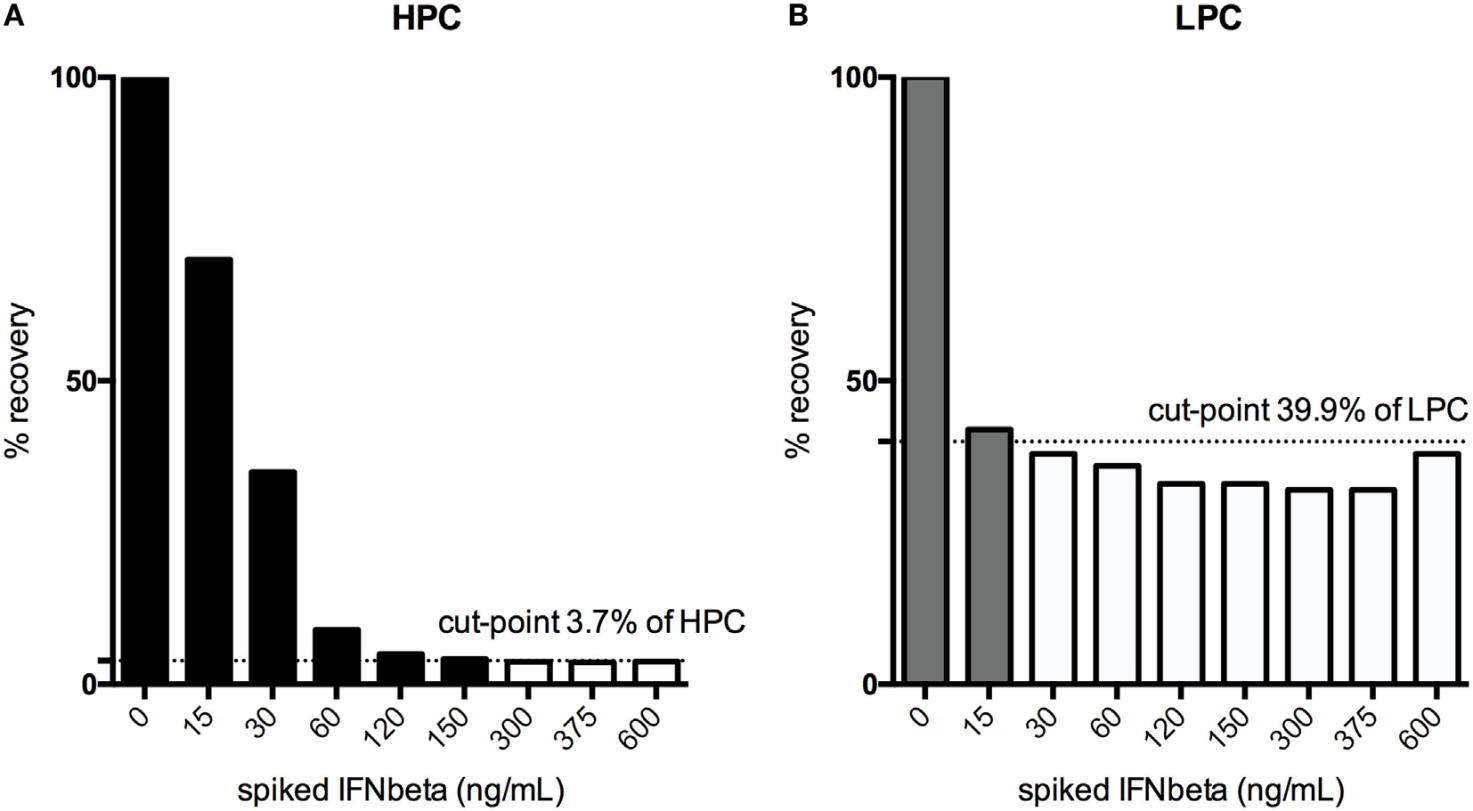
Drug interference was tested by spiking both the high-positive control (HPC), **(A)** and the low-positive control (LPC), **(B)** with increasing amounts of interferon (IFN)-β until a signal below the floating cut-point was achieved. % recovery relative to the unspiked HPC or LPC is illustrated. Black bars indicate drug concentrations that lead to a positive response, while the white bars show the drug concentrations that result in negative testing results.

For the HPC, the assay can tolerate the presence of 150 ng/mL IFN-β, for the LPC, 15 ng/mL (Figure 7).

#### Robustness

Variations in coating and blocking conditions, or sample incubation times resulting in changes to OD values below 30%, were judged acceptable. Coating times were tested overnight at 4°C (standard) and 12 days at −20°C, blocking times were extended from 60 (standard) to 90 or 120 min. Spiking times with IFN-β (0.3 µg/mL) were tested from 45, 60 (standard), and 65 min at 37°C, sample incubation times and incubation of HRP-labeled streptavidin at 55, 60 (standard), and 70 min. Incubation with biotinylated IFN-β was assessed for 55, 60 (standard), and 75 min, with optimal results for the standard time. All variations resulted in OD changes below 30%, supporting robustness of the assay.

#### Stability

The storage of samples at −20°C for a period of 3 and 6 months did not result in any significant changes of OD values compared to freshly prepared samples confirming stability of the samples for up to 6 months at −20°C.

## Conclusion

A method for the detection of binding anti-IFN-β antibodies in human serum was validated according to published recommendations (7). To reduce the false positive rate and to increase specificity, a two-tiered approach including a second confirmatory immune competition step has been developed. The assay is considered to be ultrasensitive, as it can reliably detect 26 ng/mL of the polyclonal PC of rabbit origin in human serum, which exceeds generally accepted ranges of 250–500 ng/mL in serum for antibody assays in clinical studies (15). The calculated drug tolerance for the polyclonal non-human PC antibody in this assay of 15–150 ng/mL is far above the peak serum concentration of 0.4 ng reached after an injection of, e.g., IFN-β i.m. in healthy volunteers,^3^ suggestive of the assay being insensitive to residual amounts of drug in clinical samples. Nonetheless, further studies may be warranted, as a drug tolerance assessment using the polyclonal non-human PC antibody can only partly mimic drug interference in true clinical samples, in particular, since the individual ADA repertoire may vary (11).

Overall, this assay has appropriate performance characteristics, which should be further assessed for suitability in clinical trials and clinical routine testing. Within the ABIRISK consortium, this assay will be used for testing for the development of anti-IFN-β antibodies in patient samples prospectively collected after start of therapy with IFN-β and compared with the results from bioassays used for the detection of antibodies with neutralizing capacity (8). Furthermore, newly developed and unique monoclonal anti-IFN-β antibodies of human origin will be tested, and the assay sensitivity and drug tolerance reassessed for this PC. The assay will also help to address genetic regulation of ADA responses (16–19) and prediction of immunogenicity (20, 21). The unique setting of ABIRISK, a study group that spans across different indications, will allow the comparison, for instance, of *ex vivo* phenotyping patterns with regards to their predictive value for ADA formation in several diseases, including MS, rheumatoid arthritis, and hemophilia (see text footnote 2). Improvement in methods and a deeper understanding of immunogenicity may ultimately enable us to link ADA formation to a clinical loss of response (6, 22–25), thus improving clinical care, and reducing socioeconomic costs (26, 27). We believe that ADA testing in MS may be of growing relevance, considering the recent registration of numerous alternative drugs with a varying mode of action. While these alternative treatments, such as monoclonal antibodies daclizumab, alemtuzumab, and ocrelizumab may also elicit ADA responses not yet sufficiently been studied with regards to loss of efficacy or safety, a proportion of patients with mild to moderate forms of MS will continue to be treated with IFN-β despite the occurrence of ADA and the loss of bioactivity of the drug. Considering the therapeutic alternatives that have recently become available, this should be avoided by applying reliable methods, such as the one presented here, to screen for ADA formation as part of good routine clinical practice, representing a first step toward precision medicine in MS. Such an approach is also highly relevant to the other BPs currently under development for the treatment of MS (28).

## Ethics Statement

A human serum mixed gender pool has been purchased from Sera Laboratories International Ltd., UK. Individual human serum samples derived from 10 healthy donors, and 40 IFN-β treatment naïve MS patients. The Regional Ethical Review Boards approved the use of MS patient samples, and all blood donors gave written informed consent.

## Author Contributions

Study concept and design: KI, DK, PJ, MP, BK, MR, CH, FD, PC, AF-H, and CW. Acquisition and analysis of data: KI, PJ, PC, and CW. Statistical analysis: KI, DK, and CW. Drafting of manuscript: KI and CW. Critical revision of the manuscript for important intellectual content: all authors.

## Conflict of Interest Statement

KI, PJ, CH, and MP: nothing to disclose. DK: employee of Sanofi-Aventis. MR: has received funding and speaking honoraria from Biogen Idec. FD: has participated in meetings sponsored by or received honoraria for acting as an advisor/speaker for Bayer Healthcare, Biogen Idec, Genzyme-Sanofi, Merck, Novartis Pharma, Roche, Seaton, and Teva-Ratiopharm. He is section editor of the MSARD Journal (Multiple Sclerosis and Related Disorders). TM: has received speaker honoraria from Bayer Healthcare, Biogen, Genzyme, Merck Serono, Novartis, Roche and Teva and has received travel grants from Biogen and Teva. H-PH: has received honoraria for consulting and speaking at symposia from Bayer, Biogen Idec, Genzyme, Merck Serono, Novartis Pharma, Roche, and Teva Sanofi-Aventis. BK: has received honoraria for lecturing, travel expenses for attending meetings, and financial support for research from Bayer Health Care, Biogen, Genzyme/Sanofi Aventis, Grifols, Merck Serono, Mitsubishi Europe, Novartis, Roche, Talecris, and TEVA. He is currently also an employee of Biogen. EB: an employee of Merck. PC: currently employed by UCB. AF-H: has received funding and speaking honoraria from Biogen Idec and Pfizer. CW: consulting and/or research funding: Bayer, Biogen, Novartis, TEVA.

## Footnotes

^1^https://www.imi.europa.eu/content/ongoing-projects.

^2^www.abirisk.eu.

^3^Available at: https://www.biogen.com.au/content/dam/corporate/en_AU/pdfs/products/AVONEX/AVONEX_Product_Information.pdf.
